# Neurocomputational Model of EEG Complexity during Mind Wandering

**DOI:** 10.3389/fncom.2016.00020

**Published:** 2016-03-04

**Authors:** Antonio J. Ibáñez-Molina, Sergio Iglesias-Parro

**Affiliations:** Psychology Department, University of JaénJaén, Spain

**Keywords:** neural dynamics, mind wandering, Kuramoto model, synchrony, EEG complexity

## Abstract

Mind wandering (MW) can be understood as a transient state in which attention drifts from an external task to internal self-generated thoughts. MW has been associated with the activation of the Default Mode Network (DMN). In addition, it has been shown that the activity of the DMN is anti-correlated with activation in brain networks related to the processing of external events (e.g., Salience network, SN). In this study, we present a mean field model based on weakly coupled Kuramoto oscillators. We simulated the oscillatory activity of the entire brain and explored the role of the interaction between the nodes from the DMN and SN in MW states. External stimulation was added to the network model in two opposite conditions. Stimuli could be presented when oscillators in the SN showed more internal coherence (synchrony) than in the DMN, or, on the contrary, when the coherence in the SN was lower than in the DMN. The resulting phases of the oscillators were analyzed and used to simulate EEG signals. Our results showed that the structural complexity from both simulated and real data was higher when the model was stimulated during periods in which DMN was more coherent than the SN. Overall, our results provided a plausible mechanistic explanation to MW as a state in which high coherence in the DMN partially suppresses the capacity of the system to process external stimuli.

## Introduction

Mind wandering (MW) is a transient cognitive state in which conscious attention is decoupled from the external environment of an ongoing task in favor of focusing on intrinsic, self-generated thoughts or images. This sensorial decoupling is associated with a reduction in the extent to which we process external events (Smallwood et al., 2008; Kam et al., 2010; Smilek et al., 2010; Hu et al., 2012; Mooneyham and Schooler, 2013). One immediate consequence that can be drawn from these attentional fluctuations to the ongoing task is that the same type of stimuli, presented at different times, can cause distinct effects on the dynamics of the waking brain. This study explores the idea that the consequences on brain dynamics of the presentation of a given stimuli would depend on the interplay between internally/externally oriented cognitive modes.

MW episodes are known to originate from transitions between externally guided and self-generated thoughts, and they cover about 30–50% of the waking time (Killingsworth and Gilbert, 2010). Given the pervasive presence of MW and because it seems to be hard-wired into normal human brain function (Smallwood and Schooler, 2006), there has been growing interest in this phenomenon. Despite important progress has been made, the underlying mechanism by which decoupled thoughts emerge has yet to be determined (Scott et al., 2015).

One relevant focus of research has been the neural mechanism that allows the emergence of unrelated states to the task at hand. In this line, some studies have shown that fluctuations between external attention (EA) and MW can result from dynamical interactions of distributed brain areas operating in large-scale networks (Greicius et al., 2008; Bressler and Menon, 2010). MW has been related to the activation of a default mode network (DMN) that comprises the precuneus/posterior cingulated, posterior parietal and ventromedial prefrontal cortices (Buckner et al., 2008). A high level of activation in the DMN has been found during task unrelated to experimental conditions or tasks involving internally oriented cognition such as autobiographical memory, prospection, mental navigation, and theory of mind (Spreng et al., 2010). Interestingly, when participants are focused on a specific task, the DMN is less active, while other areas of the cortex seem to participate in a higher degree (Raichle et al., 2001). Specifically, the activation of a salience network (SN) has been related to cognitive states during externally guided tasks. SN responds to the degree of subjective salience of cognitive, homeostatic, or emotional stimuli (Seeley et al., 2007).

Research should consider not only functional correlations and cognitive states, but also network interactions. In this sense, a basic observation about the interplay between the DMN and the SN is that they are reciprocally related (Fox et al., 2005; Deco et al., 2011) so that if a person is focused on an externally demanding task, the SN is more active than the DMN; on the contrary, when cognitive processes are independent of the external environment, the SN is less active than the DMN (Sharp et al., 2011). This anti-correlated pattern has led to the suggestion that the two brain networks may perform complementary or even opposing functions (Fransson, 2005). For example, the inability to suppress DMN activity can produce attentional lapses and impairs task performance (Mason et al., 2007) while DMN hyperactivity has been related to depression, anxiety, and attention deficit (Whitfield-Gabrieli and Ford, 2012). Moreover, there is evidence that the SN integrity modulates the activity of the DMN (Bonnelle et al., 2012).

Fox et al. (2005) suggested that an important aspect in the interaction between the DMN and the SN is the level of synchrony between the regions of each network. Synchronization between neural assemblies is widely accepted as a basic mechanism of communication in the brain (Lachaux et al., 1999; Varela et al., 2001; e.g., Engel et al., 2013; Marzetti et al., 2014). Functional connectivity in the DMN has been linked to power and phase synchronization between distant neural populations of the network (e.g., de Pasquale et al., 2010; Sadaghiani et al., 2010; Knyazev, 2013). In the case of the DMN and SN, because they are anti-correlated, the level of connectivity or synchrony in one network might trigger a mechanism that decreases the level of synchrony in the other network. In this line, it has been proposed that the Anterior Insula, which is part of the SN, might be responsible for an increase in the functional interplay between the networks which results in a deactivation of the DMN (see Uddin et al., 2014 for a review).

Therefore, it is reasonable to think that the functional relationship between the DMN and SN occurs through a mechanism that provides anti-correlations between their levels of internal synchrony (Uddin et al., 2009; Jilka et al., 2014). In the present study we have explored this possibility with a network of Kuramoto oscillators as a neurocomputational model. Hence, each oscillator is a functional node of the network. Note that the terms “node” and “oscillator” are indistinctly used in this manuscript. The dynamics of the model are used to simulate sets of EEGs that are compared with real EEG data. The rationale we follow is that if MW is the result of a state in which the DMN is highly synchronized and the SN is desynchronized, then, a neural model of MW with dynamics that include these characteristics will produce signals that parallels real data collected during MW. Moreover, in this paper, we compare the complexity (cortical heterogeneity or desynchronization) of simulated EEGs with the real EEGs from Ibáñez-Molina and Iglesias-Parro (2014). In the following paragraphs we present and justify the neurocomputational model we selected to construct the simulated series of EEGs.

One fruitful model to simulate synchronic behaviors in the large-scale brain networks is the Kuramoto model (Kuramoto, 1984; Strogatz, 2000, 2001). It can simulate the phase evolution of several weakly coupled oscillators that represent the mean oscillatory behavior of different cortical regions (mean-field model).

A significant development in this model was to introduce dynamics with properties of real brains (Cabral et al., 2011, 2014). In particular, connectivity between oscillators and location in space are calculated according to real structural data obtained with diffusion tensor imaging (Hagmann et al., 2008). This information allows the introduction of time delays between oscillators to simulate the effects of speed transmission between different anatomical regions. With a model of these characteristics, Cabral et al. (2011, 2014) found that a Kuramoto model produces a metastable state that can be directly related to dynamics of the DMN. Metastability, in this framework, refers to the capacity of a group of oscillators to partially synchronize their activity without become locked into a steady state. This flexible dynamics makes possible cortical oscillators to rapidly switch between different states through the reorganization of its component areas into different coordinated networks (Bressler and Kelso, 2001; Shanahan, 2010; Deco et al., 2011; Váša et al., 2015). The most used indicator of metastability in the Kuramoto networks is the variability in time (standard deviation) of the global phase coherence of oscillators. In the next sections, following Wildie and Shanahan (2012), metastability has been considered as the variability of phase coherence and has been calculated as the standard deviation of the Kuramoto order parameter *r*(t).

In a further development of the model, Hellyer et al., (2014) introduced a modification in which they manipulated the connectivity between the oscillators of the SN to simulate externally driven states. Interestingly, they found that when the connectivity between the oscillators of the SN was reinforced, the network of oscillators reached a higher level of synchrony and the metastability of the system decreased. Hence, if we accept that EA and MW are related to SN and DMN, respectively, then these findings suggest that transitions between EA and MW are characterized by changes in the general metastability of the cortex. In line with this hypothesis, Ibáñez-Molina and Iglesias-Parro (2014) found that MW states produced more irregular or complex EEG patterns than EA. In their experiment, they registered the EEG of participants while they were watching video clips, and associated long EEG segments to either MW or focused attention on the content of the videos. After that, the fractal dimension of the time series was calculated as an estimator of complexity. In this context, it is accepted that EEG complexity can be related to the amount of underlying independent neural sources and, hence, it can be sensitive to cortical synchrony (for a review, see Stam, 2005). Their results indicated that the complexity of EEGs when participants were focused on the content of the videos was significantly lower for most recording sites than complexity of EEG series associated with a MW state. These results suggest that MW arises in the presence of a heterogeneous pattern of neural activation, and similarly to what Hellyer et al. (2014) found, it might indicate that the general cortical synchrony would be reduced, when compared with signals from externally generated thoughts.

Taken together these results, we arrive to the starting point of the present work. We aim to investigate whether sets of coupled Kuramoto oscillators are able to flexibly produce outputs that can be related to either EA or MW states. To reach this goal, we have explored the structural similarity between simulated EEGs obtained with the model and real EEGs during externally and internally generated thoughts using EEG data sets from Ibáñez-Molina and Iglesias-Parro (2014).

**Figure 1:**
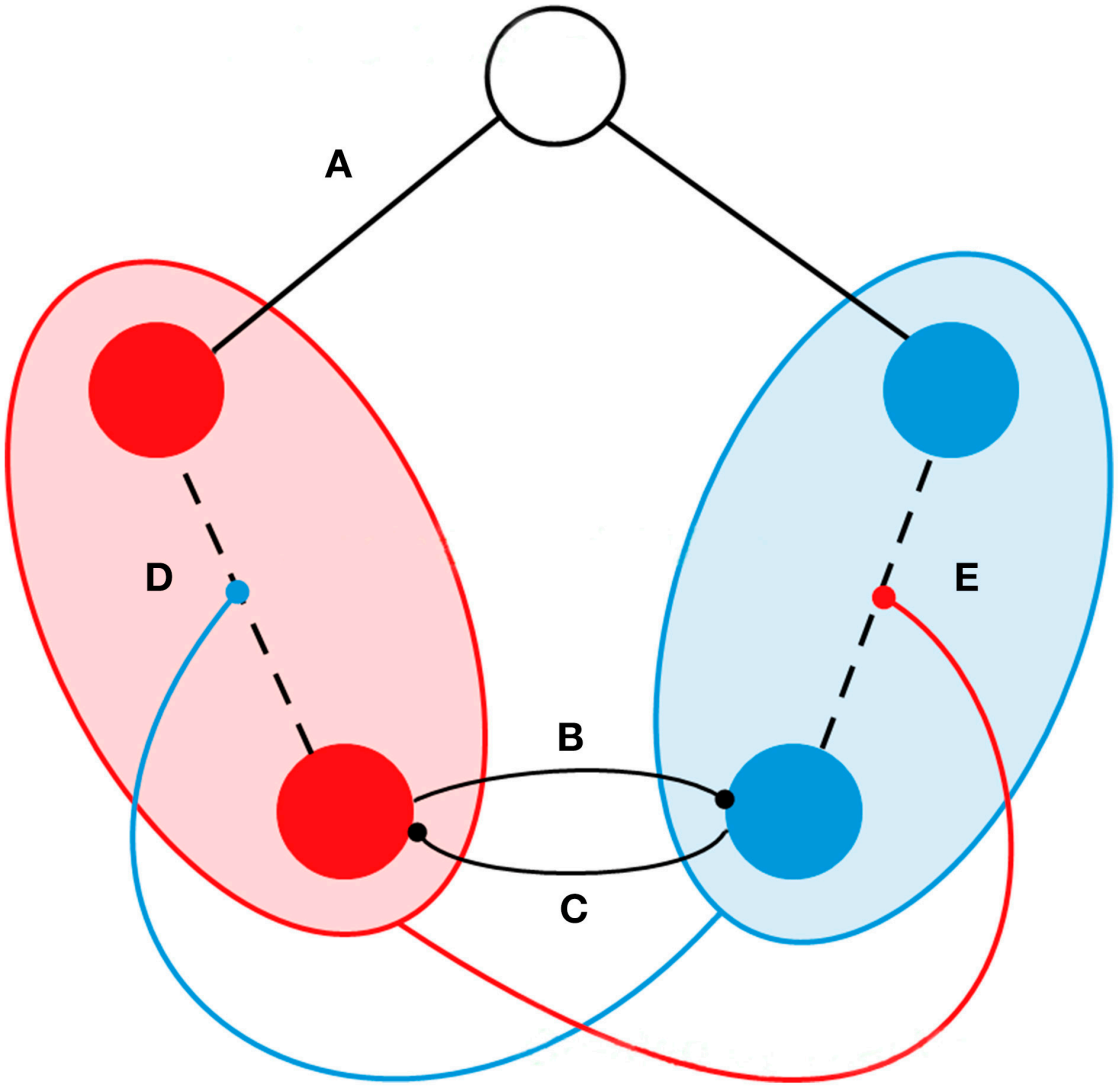
**Schematic representation of the functional interactions in the model**. Circles represent examples of five oscillators/nodes. SN and DMN are represented by red and blue respectively. The node in white indicates that it does not belong to the SN or DMN. We introduced the following types of dynamical interactions: **(A)** If the node does not belong to the SN or the DMN, the connectivity was fixed during simulations and it corresponded to the structural connectivity *C*_*ij*_. **(B)** The phase of nodes from the DMN competed with nodes from the SN. **(C)** The phase of nodes from the SN competed with nodes from the DMN. **(D)** Connectivity between nodes in the SN is proportionally weakened by the coherence of the DMN. **(E)** Connectivity between nodes in the DMN is proportionally weakened by the coherence of the SN.

As in Hellyer et al. (2014), we considered that, when a stimulus is presented to the system, the SN will increase its internal connectivity and the model will switch to a different state. However, Hellyer did not explicitly take into account the competing dynamics between the DMN and the SN. As we mentioned above, both networks tend to be anti-correlated even in resting conditions; and then, the modifications we introduced in the model were based on this experimental observation. In particular, the level of synchrony in one network at time *t* is taken as a variable to reduce the internal connectivity of the competing network, and the interconnectivity between the oscillators of both networks (see Figure 1). For example, if the synchrony in the DMN is very high, the model will tend to reduce the connectivity in the SN and between the nodes of the DMN and SN. In our study, we took these self-regulated dynamics as the baseline of the model.

In a second step, we simulated the presentation of external events by means of an increase in the internal connectivity of the SN. Experimental evidence has shown that one of the functions of the SN is to facilitate the processing of external events. Once a stimulus reaches the cortex, the SN activates a process of identification and executive functions directed to establish externally directed attention. In this process, the SN also deactivates the DMN (Sridharan et al., 2008; Menon and Uddin, 2010). Hence, it is possible to introduce physiologically plausible external stimulation to the model by a transient reinforcement of the SN connectivity.

This modified model permits to simulate the presentation of stimuli in two different critical conditions. First, to simulate external stimulation when oscillators from the SN are more synchronized than those from the DMN. The other option is to model the presentation of stimuli when the internal synchrony of the DMN is above the level of synchrony in the SN. This distinction is important because we assume that, when the SN is more synchronized, the state of the system will parallel EA. Then, any stimulus presented during this state will be expected to produce a pattern that can be related to external awareness of events. On the contrary, if DMN exhibits higher synchrony than the SN, the system will be in an internally driven mode, and external events will drive the dynamics of the model toward a state that resembles MW. Then, the predictions from our model will be:

- The Simulation of the EA condition will produce more globally synchronized states than MW. Stimulation when the SN is less synchronized than the DMN will result in dynamics with low global synchrony. On the contrary, stimulation when the SN is more synchronized, then the DMN will drive the system to dynamics with high global synchrony.
- The complexity of the simulated EEGs will correspond to the complexity of real data in Ibáñez-Molina and Iglesias-Parro. Lower levels of complexity are expected in most sites for the externally driven state of the model when compared with the MW state.

## Methods

### The Kuramoto model

The model for MW consisted in a set of differential equations in the following form:

$$\frac{d\theta_{i}}{dt}=\;\omega +k\underset{j=1}{\sum^{N}}a_{ij}c_{ij}\sin\left(\theta_{j}\left(t-\tau_{ij}\right)-\theta_{i}(t)\right),\;i=1,\ldots ,N.$$

The Kuramoto model has been used to simulate synchronized behaviors in a wide variety of domains. Here we have adapted the version from Cabral et al. (2011). These authors were the first to introduce time-delays between brain areas in a Kuramoto network. The model defines the dynamics of a network with 66 oscillators (or nodes) coupled together according to human white matter tractography (Hagmann et al., 2008). Each node represent a cortical region or the brain located in a three dimensional space. The length and fiber density served as the basis for the elaboration of the connection strength (*c*_*ij*_) and conduction delays (τ_*ij*_) between oscillators. These matrices have been widely reported and might be available upon request (Hagmann et al., 2008). Θ_*i*_ is the phase of the i*th* oscillator on its limit cycle and ω is its natural frequency in radians (60 Hz). The control parameter *k* is the global excitatory coupling strength, a parameter that scales all coupling strengths. *N* is the total number of oscillators.

Finally, *a*_*ij*_ is a dynamical modifier of the connectivity between oscillators ranging from 0 to 1. *a*_*ij*_ take values that are proportional to the degree of synchrony of the SN and DMN. For a Kuramoto model, the degree of synchrony between oscillators is conveniently measured by an order parameter (*r*(t)) that satisfies:

$$r(t)e^{i\psi }=\frac{1}{N}\underset{j=1}{\sum^{N}}e^{i\theta_{j}},$$

where 0 ≤ *r*(t) ≤ 1 measures the phase coherence of the *N* oscillators population (henceforth *r*(t) and phase coherence will be used as synonyms), and ψ is the average phase (Acebrón et al., 2005). Critically, *a*_*ij*_ depends on the phase coherence of the SN and DMN sub-networks. The order parameter is calculated over the SN and DMN networks separately and defined as *r*_*sn*_(t) and *r*_*dmn*_(t), respectively. Hence, *a*_*ij*_ is a matrix that change in time and it follows the rules:

$$a_{ij}=\;\{\begin{array}{cc} 1, & if\;\;i\;and\;j\;\notin \;\{SN,DMN\}\\ -r_{sn}\left(t-\tau_{ij}\right)b, & i\;\in \;\left\{DMN\right\},\;\;and\;j\;\in \;\left\{SN\right\}\\ -r_{dmn}\left(t-\tau_{ij}\right)b, & if\;\;i\;\in \left\{SN\right\}\;\;and\;\;j\;\in \;\left\{DMN\right\}\\ 1-r_{sn}\left(t-\tau_{ij}\right)d,\; & if\;i\;\;and\;j\;\in \left\{DMN\right\}\\ 1-r_{dmn}\left(t-\tau_{ij}\right)d, & if\;i\;\;and\;j\;\in \left\{SN\right\} \end{array}$$

where *b* and *d* are scaling parameters. Rules for the dynamics of *a*_*ij*_were selected to match the above mentioned experimental observations. The level of synchrony in one network is taken as a variable to reduce the internal connectivity of the competing network (terms −*r*_*sn*_(*t* − τ_*ij*_)*b* and −*r*_*dmn*_(*t* − τ_*ij*_)*b*), or to modulate the interconnectivity between the two networks (terms 1−*r*_*sn*_(*t* − τ_*ij*_)*d* and 1−*r*_*dmn*_(*t* − τ_*ij*_)*d*). For example, one oscillator from the SN reduces its connectivity with each oscillator of the DMN proportionally to *r*_*dmn*_(*t* − τ_*ij*_) and modulates its connectivity with each oscillator of the SN proportionally to *r*_*sn*_(*t* − τ_*ij*_).

Following Hellyer et al. (2014), each sub-network consisted of six key oscillators (three from each hemisphere). Oscillators of the SN were located in the superior parietal cortex, pars opercularis and superior frontal gyrus. Oscillators of the DMN were located in the inferior parietal cortex, rostral anterior cingulate cortex and isthmus of the cingulate cortex.

There are only four free parameters in our model. The values of these parameters were selected so that the global dynamics exhibited high metastability (standard deviation of global phase coherence) and anti-correlations between the SN and DMN. For ω = 60 Hz (gamma rhythm), these two conditions were evident with $\hat{\tau }$ = 3.5 ms, *k* = 10, *b* = 0.5, and *d* = 1. Here, $\hat{\tau }$ refers here to the mean value of τ_*ij*_. Note that any change in $\hat{\tau }$ can be considered a change in the mean velocity of the conduction delays between oscillators. However, it is important to note that other parameter configurations produced similar behaviors in the model. For example, we found approximately the same effects for the 2.5 < $\hat{\tau }$ < 5 and 8 < *k* < 12 ranges.

**Figure 2:**
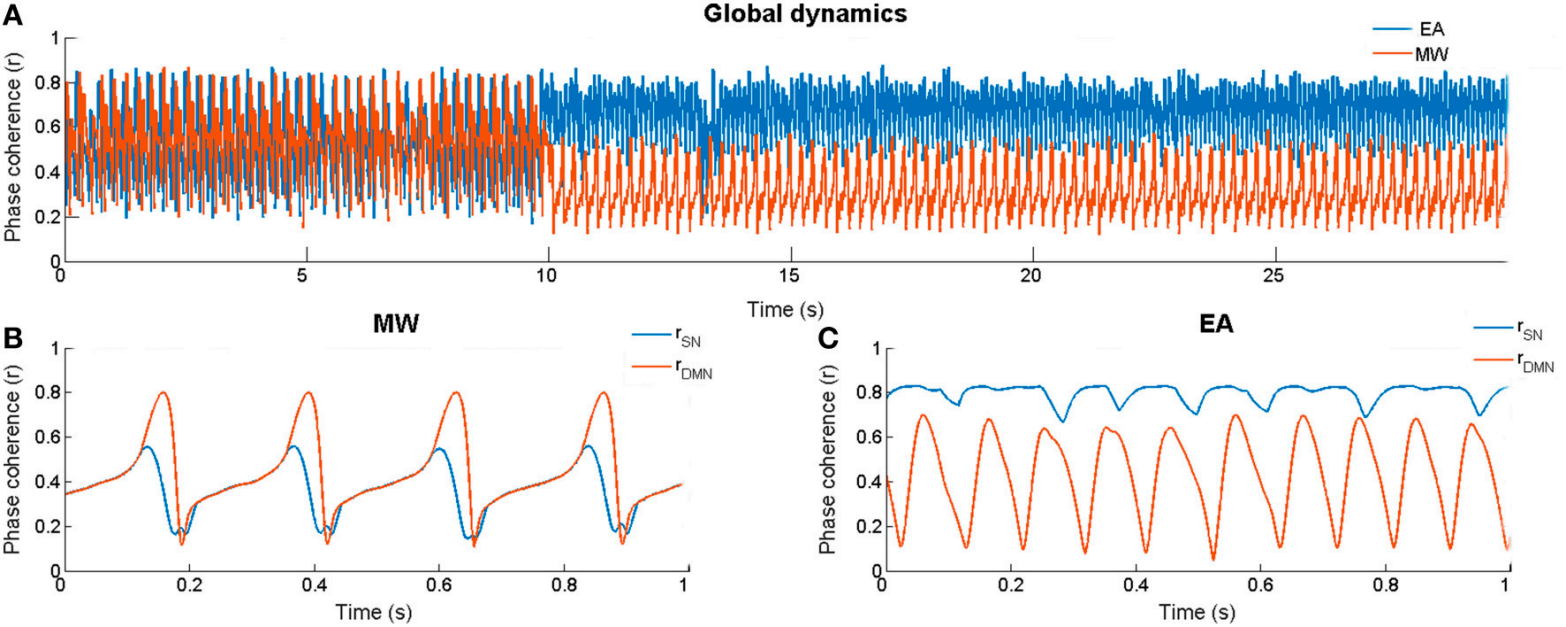
**Dynamics of the model. (A)** Global phase coherence (*r*) for External Attention (*r*_dmn_(t) < *r*_sn_(t)) and Mind Wandering (*r*_dmn_(t) > *r*_sn_(t)) in one simulation run (10 s of baseline followed by 20 first s of the stimulation period). **(B)** Phase coherence for the DMN and the SN during a section of stimulation (magnification from s 28) when *r*_dmn_(t) > *r*_sn_(t). **(C)** Phase coherence for the DMN and the SN during a section of stimulation (magnification from s 28) when *r*_dmn_(t) < *r*_sn_(t).

The Kuramoto model was simulated ten times for each condition (EA and MW). Each simulation consisted of a baseline of 10 and 50 s in which the SN was stimulated to produce EA or MW (see Figure 2A). As in Cabral et al. (2014), we used an Euler scheme in which the time step of numerical integrations was set to 0.1 ms. In Figures 2B,C, we magnify two segments of the post stimulation period for the MW and EA conditions respectively. In each of these panels we show the phase coherence dynamics for the SN and for the DMN.

### Simulation conditions for EA and MW

In order to simulate EA and MW states in the model, it is necessary to introduce external perturbations that represent sensory stimuli. In the present work we operationalized the activation of a specific cognitive state by increasing the connectivity strength between the nodes of the SN. Specifically, this was conducted by multiplying these connectivity values in c_*ij*_ by a specific magnitude (*s*).

We simulated external perturbations in the model in two conditions:

a) EA stimulation:

sc_*ij*_ when *i* and *j* oscillators ϵ {SN} and r_*dmn*_(t) < r_*sn*_(t)

b) MW stimulation:

sc_*ij*_ when *i* and *j* oscillators ϵ {SN} and r_*dmn*_(t) > r_*sn*_(t)

where, *s* represents the strength of the external stimulation and *c*_*ij*_ is the connection strength matrix.

Thus, the difference between EA and MW simulations critically depended on the time period at which the stimulation was presented. We rationalized that, when coherence in the SN was higher than in the DMN, stimulation would result in an EA-like state. However, when coherence in the SN was lower than in the DMN, stimulation would produce a MW-like state.

In our simulations, *s* = 10 and it modeled the strength of external stimulation. Hence, stimulation periods were simulated on each condition by changing c_*ij*_ by sc_*ij*_ when the aforementioned inequalities where found. In the first versions of our model, EA inequalities were met much more frequently than those of MW. Obviously, it resulted in longer stimulation periods for EA than for MW. To set statistically equivalent time periods of stimulation in both conditions, we introduced discrete stimuli in which a maximum stimulus length was set to 100 ms. Additionally, we provided with an after stimulus refractory period for each condition, which was set to 100 ms for EA and 0.5 ms for MW states. Mean values and metastability of *r*(t), *r*_*sn*_(t), and *r*_*dmn*_(t) were the dependent variables. As stated before, the degree of metastability was calculated as the standard deviation of these parameters.

### EEG simulations

EEG activity from 32 sensors was simulated for each model according to the following weighted sum of the activity in each source (oscillator):

$$x_{i}(t)=\sum_{j=1}^{N}w_{ij}\sin\left(\theta_{i}(t)\right)+\varepsilon_{i}\left(t\right),\;i=1,\ldots ,P,$$

where *x*_*i*_(t) is the time series from sensor i*th*, and *w*_*ij*_ is the weighted contribution of oscillator j*th* in sensor i*th*. Each *w*_*ij*_ was calculated using a standard forward model algorithm implemented in the software Besa 2000 (see Hoechstetter et al., 2004). The model provides a solution of the projection of the functional sources (oscillators) on the sensors distributed on scalp. The contribution of each oscillator to each sensor was obtained in two steps. First, each oscillator was located in a head model according with its Talairach coordinates. After this step, each oscillator is considered a cortical source with a particular contribution to each sensor. This contribution depended on the forward model implemented in the software. Second, the weights of these sources were normalized for each sensor to a maximum value of 1. This matrix is available upon request. The term ε_*i*_(t) represents uncorrelated white Gaussian noise with 20 dB SNR added to the signal. The resulting EEG series were used as inputs for a custom Matlab script developed for the calculation of fractal complexity (Higuchi Fractal Dimension, HFD) of the signals. HFD is a measure of irregularity for discrete time series. The algorithm obtains new series by sampling the original signal at different intervals (*k*). For each *k*, the lengths, *L*(*k*), of the signals are calculated normalizing the sums of the differences of the values, with a distance of *k* and a starting point *m* (*m* = 1, 2,…, *k*). Finally, a double logarithmic plot, ln *L*(*k*) vs. ln *k*, is used to estimate the actual dimension value of the signal. The range of values for HFD lies between 1 and 2. Dimensions close to 1 correspond to simple curves such as a sinusoidal wave, and values close to 2 correspond to signals with randomly distributed values (Higuchi, 1988). Values of HFD were obtained for each EEG epoch using a 128 ms sliding window with 13 ms of time overlapping. The scaling parameter kmax was set to 18. The series of HFD values we obtained for each segment were averaged across sensors. These values were averaged across simulations in each condition for statistical analyses.

## Results

Since the dynamics of the model were expected to be complex and variable on each realization, we analyzed the results using standard statistical approaches of correlation and analysis of variance. We provide between brackets the values of statistical estimators and its associated probability.

### General dynamics of the model: Baseline

As can be seen in Figure 2A, our model was highly metastable in its baseline, showing variations in *r*(t) ranging almost all possible values (approximately from 0.2 to 0.8). To test if DMN and SN were anti-correlated, Spearman rank order coefficient was calculated between phase coherence of the DMN and the SN (*r*_dmn_(t) and *r*_sn_(t) respectively). The first 1500 samples were eliminated because they reflected the influence of the initial conditions. Series were windowed (1000 samples) and Spearman was computed for each non-overlapping window. Thus, for each of the series we obtained 98 correlation coefficients. In order to obtain a more stable estimation of ρ, Spearman coefficients were averaged across the simulations and, next, the 98 obtained coefficients were averaged. Averages were made using the Fisher transform (Fisher, 1973; Corey et al., 1998). We found a significant negative correlation between networks (*rs* = −0.396; *p* < 0.01).

### Comparisons between baseline and stimulation periods

In order to compare the global behavior of the model between baseline and stimulation periods, paired *t*-tests were performed, with the mean and the standard deviation of phase coherence of simulations as dependent variables. The mean and the standard deviation of phase coherence were obtained from the entire segments (during baseline as well as during post stimulation period) and averaged across simulations. Obtained results showed that, during EA, *r*(t) mean increased during stimulation [*t*_(9)_ = −39.93; *p* < 0.01]. However, in MW, *r*(t) mean significantly decreased after stimulation [*t*_(9)_ = 223.05; *p* < 0.01]. The variability of *r*(t) decreased after stimulation during EA [*t*_(9)_ = 49.87; *p* < 0.01] as well as in MW [*t*_(9)_ = 44.32; *p* < 0.01]. Taken together, these results indicated opposite effects of stimulation on the mean phase coherence (an increase of coherence in EA and a decrease of coherence in MW) accompanied by a global reduction of the metastability (standard deviation of phase coherence).

**Figure 3:**
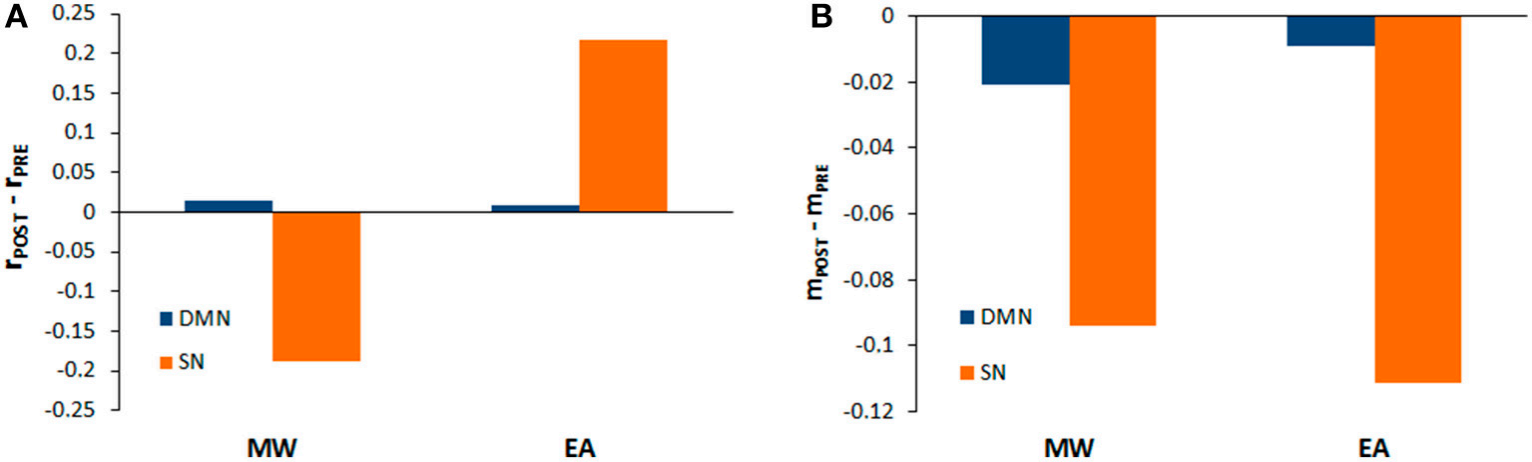
**Phase coherence mean and variability during stimulation periods. (A)** Mean of the phase coherence (r_POST_ – r_PRE_) for the DMN and the SN when *r*_dmn_>*r*_sn_ (MW) and *r*_dmn_<*r*_sn_(EA). **(B)** Phase coherence variability (m_POST_ – m_PRE_) for the DMN and the SN when *r*_dmn_>*r*_sn_ (MW) and *r*_dmn_<*r*_sn_(EA).

For each condition (EA vs. MW), we also compared the behavior of each sub-network (SN and DMN) in the transitions between baseline and stimulation. To do this, for EA as well as for MW, the pre-post stimulation behavior of SN and the DMN were analyzed by paired *t*-tests using the mean (see Figure 3A) and the standard deviation of phase coherence (see Figure 3B) of simulations as dependent variables.

In the EA condition, we found a significant increase in the mean of phase coherence of SN after stimulation [*t*_(9)_ = −103.08; *p* < 0.01] as well as in the mean of phase coherence of DMN [*t*_(9)_ = −14.73; *p* < 0.01]. On the other hand, in the MW condition, the mean of the phase coherence of the SN decreased significantly after stimulation [*t*_(9)_ = 549.79; *p* < 0.01] but the mean of the phase coherence of the DMN increased significantly after stimulation [*t*_(9)_ = −403.65; *p* < 0.01]. These analyses allow us to qualify the previous results of the global model. Thus, stimulation produced an increase in coherence during EA both for the DMN and for the SN, but there was a dissociation in phase coherence during MW after stimulation. Specifically, although stimulation increased coherence for the DMN, the stimulation reduced coherence for the SN.

Regarding the variability of phase coherence, we found a significant reduction in metastability after stimulation, both for SN and DMN. Specifically, in the EA condition, we found a significant reduction in variability for the SN and DMN after stimulation [*t*_(9)_ = 29.94; *p* < 0.01; *t*_(9)_ = 18.62; *p* < 0.01, respectively]. Similarly, in the MW condition, both the SN and DMN variability decreased after stimulation [*t*_(9)_ = 204.63; *p* < 0.01; *t*_(9)_ = 116.20; *p* < 0.01, respectively].

### Comparisons between EA and MW in stimulation periods

In order to have a closer picture of the global behavior of the model during the stimulation, *t*-test were conducted to compare the mean phase coherence and the variability of phase coherence of EA and MW. Thus, when *r*(t) was compared for EA and MW during stimulation, *r*(t) was significantly higher in EA than in MW [*t*_(18)_ = 752.06; *p* < 0.01]. The variability of phase coherence was significantly lower in EA than in MW [*t*_(18)_ = −1057.76; *p* < 0.01]. Thus, during stimulation in EA, the system globally showed an increase in coherence and a reduction of the metastability.

When the SN and DMN are analyzed separately, different dynamics were found for EA and MW conditions. During EA, the obtained results showed that *r*(t) was significantly higher in the SN than in the DMN [*t*_(18)_ = −201.53; *p* < 0.01; see Figure 3A]. However, the variability of phase coherence was significantly lower in the SN than in the DMN [*t*_(18)_ = 30.48; *p* < 0.01; see Figure 3B]. On the contrary, when the system was set in a MW state, coherence was significantly lower in the SN than in the DMN [*t*_(18)_ = 752.06; *p* < 0.01]. Finally, variability of phase coherence was significantly lower in the SN than in the DMN [*t*_(18)_ = 1057.76; *p* < 0.01]. Hence, when stimulation occurred in EA states, coherence was higher for SN, but when stimulation occurred during MW, coherence was higher for DMN. Interestingly, during stimulation, the SN showed a lower metastability than the DMN in both conditions (EA and MW).

### Comparisons between EEG simulations and experimental data

In order to compare simulated and empirical data, a measure of signal complexity, Higuchi fractal dimension (HFD), was calculated from EEG data. The HFD mean was obtained from empirical dataset for the EA condition on each channel (*M* = 1.47, *SD* = 0.02) and for MW (*M* = 1.53, *SD* = 0.03). In a similar way, HFD was calculated from simulated data during EA (*M* = 1.81, *SD* = 0.05) and MW (*M* = 1.90, *SD* = 0.05).

With the aim to compare the empirical HFD during EA with the HFD in MW an ANOVA was conducted. Obtained results [*F*_(1, 58)_ = 59.89; *p* < 0.01] showed that complexity was significantly higher during MW. Same analysis conducted with simulated data revealed the same data pattern [*F*_(1, 58)_ = 40.94; *p* < 0.01].

**Figure 4:**
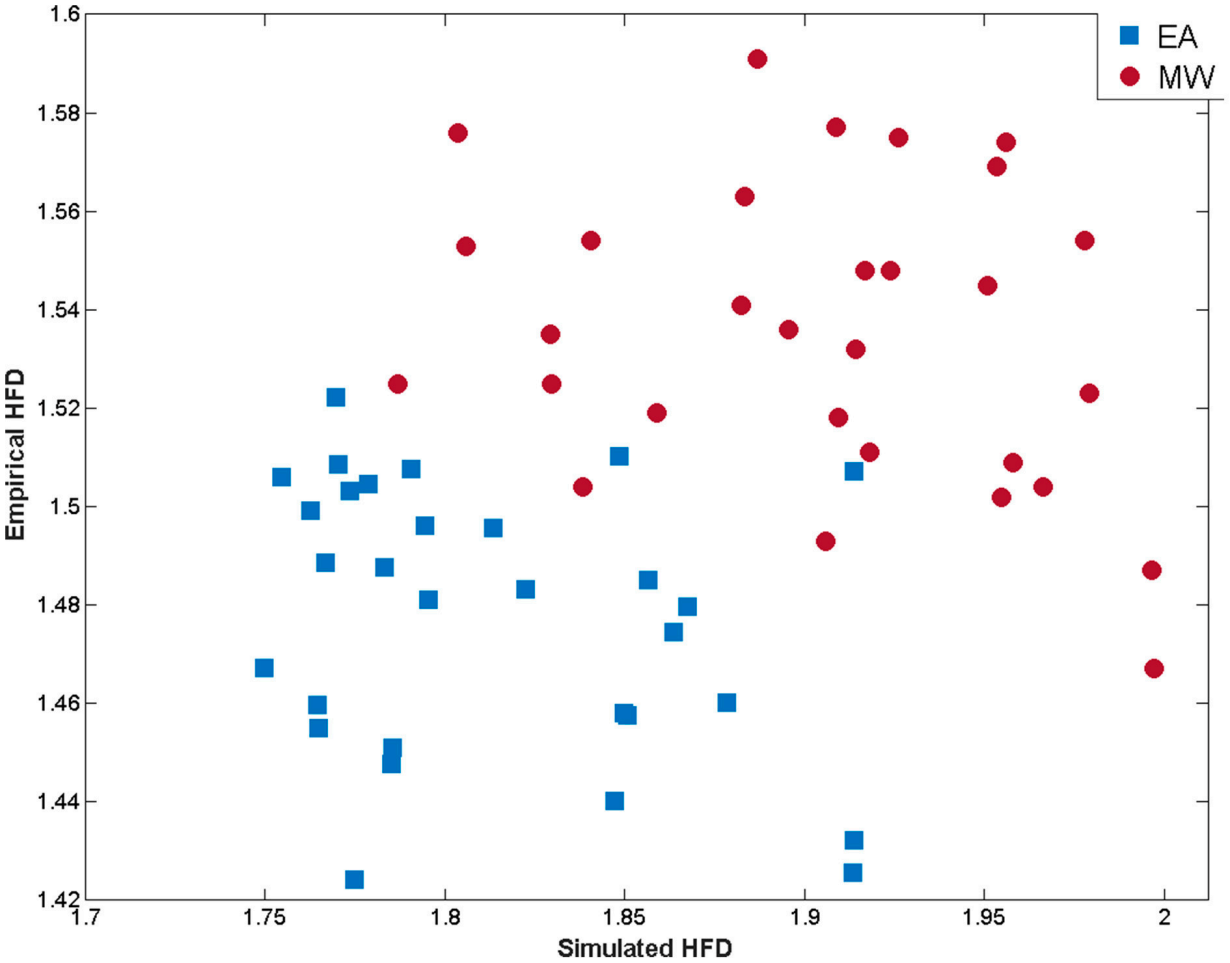
**Scatterplot of empirical and simulated measures of signal complexity (HFD) at each EEG channel for EA (blue squares) and MW (red dots)**.

Furthermore, a discriminant analysis was conducted to predict whether a cognitive state was EA or MW. Predictor variables were the empirical and simulated cortical HFDs. In order to check the hypothesis that the covariance matrices do not differ between EA and MW groups, a Box's M test was performed. Results indicated that the statistical assumption of equality of covariance matrices was met (*M* = 1.10; *p* = 0.78). A canonical correlation of 0.842 suggests that the linear combination of empirical and simulated HFD explains the 70.89% of the variation in the cognitive state (i.e., EA vs. MW, see Figure 4). The Wilks' lambda (λ = 0.29; *p* < 0.01) indicated that the proportion of variance explained by the model is significant. The standardized canonical discriminant function coefficients (0.88 for empirical HFD and 0.79 for simulated HFD) indicated that both predictors were almost equally good but independent predictors of group membership. Following the suggestion of an anonymous reviewer, we repeated the analysis, but using the empirical HFD as the only predictor. In this case we found an explained variance of 50.40%. This reduction in explained variance may indicate that the inclusion of simulated data improves the classification of cognitive states beyond that obtained by considering only empirical HFD.

The cross-validation results using a jack-knife process of classification revealed a hit ratio of 95%. That is, the 95% of channels were classified correctly into EA or MW conditions. External attention episodes were classified with better accuracy (96.7%) than MW ones (93.3%).

## Discussion

In this study, we wanted to explore how the brain effect of a perturbation depends on the timing of synchrony between networks. We have proposed a large-scale neural network model to simulate the complexity of brain signals generated during EA and MW. In this vein, we introduced a Kuramoto network of coupled oscillators to model the activity of large cortical areas during EA and MW states. The parameters of the model were adjusted so that the general phase coherence of the oscillators before stimulation exhibited a metastable state. In addition, nodes belonging to the SN and DMN were allowed to compete according with the level of coherence in each cluster. This condition was introduced in agreement with experimental evidence showing that the activation of the SN and DMN is anti-correlated in an off-task context (Fox et al., 2005; Buckner et al., 2008; Sharp et al., 2011; Jilka et al., 2014). EA and MW were simulated by transient increases in the connectivity of the SN. Specifically, MW was simulated by increasing the connectivity of the SN when *r*_dmn_(t) was higher than *r*_sn_(t), and externally focused attention was simulated by increasing the connectivity in the SN when the *r*_dmn_(t)was lower than the *r*_sn_(t). Simulated EEGs obtained with output phases of the nodes in the Kuramoto network for both conditions, revealed the same complexity patterns (HFD) in EA and MW than the real EEG registered from participants.

This neurocomputational model exhibited metastable coherence between the nodes representing each cortical area during the resting state phase. This metastability is considered essential to information processing and communication (Yang et al., 2012) and to subjective cognitive states and consciousness (Hudetz et al., 2014). Moreover, metastability was a necessary condition to achieve a state that resembled a resting state function of the brain (see Cabral et al., 2011, 2014; Hellyer et al., 2014). To obtain a metastable state of the system, we adjusted the parameters for the mean time delay and the global coupling between oscillators.

The other key feature of the resting state is that coherence values in the SN and the DMN are anti-correlated. We took into account this experimental observation and modified the Kuramoto model to capture the effect. The connectivity between a given pair of nodes was allowed to change according to the coherence of the sub-network where they belong. To the best of our knowledge, this is the first time that coherence values, here *r*(t), from sub-networks are used as inputs to regulate the functional connectivity in the dynamics of the model. By using this procedure, we overcame one of the caveats of the models proposed so far, which is that connectivity between each pair of nodes depends on fixed and structure-based parameters (for a review see Nakagawa et al., 2013). Therefore, our baseline network model showed patterns of anti-correlation between the SN and DMN. The understanding of the mechanisms that generate the dynamics of complex systems is important because it can provide useful information regarding the functional characteristics of the brain. In the present paper we have tried to offer a fine-grained description of global dynamics of the model using the behavior (phase coherence mean and variability) of the SN and DMN sub-networks. In this regard, anticorrelation between SN and DMN sub-networks during resting state was an important feature of the model because it causes a high competitive state between the different sub-networks that are involved in different functions or tasks (Deco et al., 2009). These dynamics resembles what Shanahan (2010) or Wildie and Shanahan (2012) called chimera states, characterized by coexistent synchronized and desynchronized subsystems.

Important for our goals, external inputs presented to the system produced different outcomes depending on the relative strength of the connections in the SN and DMN. First, when the SN was more coherent than the DMN, an increase in the SN connectivity resulted in a global enhancement of the *r*(t), in which the *r*_sn_(t) was higher than the *r*_dmn_(t). This result is equivalent to those reported by Hellyer et al. (2014) using a similar model and approach. They found a match between simulated data and results from an experiment where fMRI was registered during an attentional demanding task. Second, when we simulated the appearance of an external event during more coherent states of DMN relative to SN, we found a reduction in the global synchrony of the system, *r*(t). Additionally, even though the SN connectivity was transiently increased, *r*_sn_(t) showed a significant reduction relative to the *r*_dmn_(t). This surprising effect indicated that when the DMN is more coherent than the SN, the entire system is in a state in which a sudden reinforcement of the sub-network that process external events cannot easily overcome the functional dominance of the DMN. Furthermore, as shown in Figure 2B, *r*_dmn_(t) remained higher than the *r*_sn_(t) which tended to be higher even in the baseline dynamics of the simulations. Since the coherence of the DMN persisted after sequential reinforcements of the SN in this condition, we can conclude that the simulations presented here share the main functional characteristics of a MW state, in which external events are superficially processed and their presentation does not seem to challenge the dominance of the DMN. Then, our results are in agreement with the finding of a reduced processing of external events during DMN activation (sensorial decoupling). For example, Smallwood et al. (2012) reported that when the BOLD signal was high in regions of the DMN, actions related to external events were slower than actions that were not based on perceptual inputs. Similarly, Baird et al. (2014) have shown reduced inter trial coherence during MW when compared with focused attention. These authors suggested that, during MW, the DMN is active and the cortex is not entirely ready to process external stimuli.

Importantly, the results of the simulations we report are supported by experimental data obtained in our lab from EA and MW states (Ibáñez-Molina and Iglesias-Parro, 2014). We found consistent electrode-by-electrode correlation between real and simulated data. Specifically, the complexity of the EEGs registered when participants reported to be in a MW state resembled those of the simulated EEGs when the system was perturbed during a period in which the *r*_dmn_(t) was higher than *r*_sn_(t). Moreover, the complexity of the EEGs during EA was similar to the complexity of the EEGs generated from the network when *r*_dmn_(t) was lower than *r*_sn_(t). The comparison between the model and experimental results helps to understand why the complexity of the EEGs during MW is high for most electrodes distributed across the scalp when compared with the complexity obtained during EA. EEG complexity, measured as irregularity in the structure of the series, has been related to the amount of independent cortical generators (e.g., Lutzenberger et al., 1995; Escudero et al., 2015). Thus, well-integrated cortical patterns of activity would be associated with lower values of complexity than less organized cortical activation. This led Ibáñez-Molina and Iglesias-Parro (2014) to conclude that MW consists in a state in which cortical generators are diverse and the resulting EEG signals are highly irregular. In this study, we are able to qualify this source of irregularity because we found that the simulated data that better matched EEG data in MW were those from stimuli presentation when *r*_dmn_(t) was higher than *r*_sn_(t). Then, we suggest that when the activity of the DMN is dominant, the global activity of the system might consist of cortical activity with reduced coherence that produces irregular patterns of brain signals. This is in line with other observations reported in the literature. For example, Mayhew et al. (2013), found that the high cortical variability at rest, when the DMN is more active, was reduced during the presentation of simple visual and auditory stimuli. When the DMN is active, the activity of the cortex is irregular when compared with on task conditions.

Simulation is a powerful tool that provides important insights about the relationship structure-function of the brain. However, as it is necessary to simplify brain structure and dynamics, the scope of our findings has limitations. The computational model we used (a variant of Kuramoto model to take into account conduction delays) has showed rather relevant research results (Breakspear et al., 2010; Cabral et al., 2011, 2014). However, as a model, is a simplified representation of a complex dynamical system. In that sense, others abstractions are also possible such as Wilson–Cowan or Lotka–Volterra models. The use of a low-dimensional connectivity matrix, in which the SN and DMN are identified with specific groups of nodes, is far from real neural networks. In the future, it will be important to develop more realistic connectivity matrices with more nodes representing additional structures in the brain. From a functional point of view, one limitation in our study is that we took into consideration only two sub-networks and it would be desirable to explore the interactions between more sub-networks into the general network. Finally, future work should examine multi-frequency interactions between nodes arising from the models.

## Author contributions

All authors listed, have made substantial, direct and intellectual contribution to the work, and approved it for publication.

### Conflict of interest statement

The authors declare that the research was conducted in the absence of any commercial or financial relationships that could be construed as a potential conflict of interest.
